# Editorial: Pharmaceutical policy, impact and health outcomes

**DOI:** 10.3389/fphar.2023.1150055

**Published:** 2023-02-14

**Authors:** Hye-Young Kwon, Brian Godman

**Affiliations:** ^1^ Division of Biology and Public Health, Mokwon University, Daejeon, Republic of Korea; ^2^ Department of Public Health Pharmacy and Management, School of Pharmacy, Sefako Makgatho Health Sciences University, Pretoria, South Africa; ^3^ Strathclyde Institute of Pharmacy and Biomedical Sciences, University of Strathclyde, Glasgow, United Kingdom; ^4^ Centre of Medical and Bio-Allied Health Sciences Research, Ajman University, Ajman, United Arab Emirates

**Keywords:** pharmaceutical policy, non-communicable diseases, cancer, cost-effectiveness, polypharmacy, procurement policies, impact, health outcomes

Pharmaceutical policy is essential given increasing expenditure on medicines and only finite resources, with global expenditure on medicines estimated to reach US$1.5 trillion by the end of 2023 (IQVIA, 2019). This represents an annual compounded growth rate of 3–6% a year in recent years, driven by increasing prevalence of non-communicable chronic diseases (NCDs) with ageing populations and resultant increase in medicine use alongside the increasing costs of new medicines especially for orphan diseases and cancer (Godman et al., 2018; Luzzatto et al., 2018; Godman et al., 2021a; Godman et al., 2021b). The cost of cancer care is a particular Research Topic with world-wide sales of oncology medicines expected to reach $237 billion by 2024, and continue growing (Godman et al., 2021b). This increase is driven by the increasing prevalence rates of patients with cancer alongside the increasing costs of new oncology medicines, with requested prices per life year gained for new oncology medicines rising four-fold or more during the past years after adjusting for inflation (Godman et al., 2021b). This increase is exacerbated by the emotive nature of the disease area (Haycox, 2016). These growth rates though are unsustainable leading to greater scrutiny over the cost and value of new oncology medicines, which will continue (Godman et al., 2021b).

Alongside this, there needs to be greater scrutiny over the costs and use of existing medicines to ensure maximum value, with more than half of all medicines prescribed or dispensed inappropriately (Godman et al., 2021a). In addition, health authorities may not always realise the full potential for available savings. This happens when there are limited demand- and supply-side measures to encourage the preferential prescribing of low cost multiple-sourced medicines or biosimilars as seen in South Korea compared with Western European countries including Sweden and the UK (Kwon and Godman, 2017; Kim et al., 2020; Godman et al., 2021a). The ideal is that patients receive medicines appropriate to their clinical needs, in doses and duration cognisant of their requirements, and at the lowest cost to them and the health service. However, this is not always the case. Alongside this, we are aware there are concerns with rising rates of antimicrobial resistance (AMR), increasing morbidity, mortality, and costs (Godman et al., 2021c). This results from excessive use of antibiotics especially for self-limiting conditions alongside patients with tuberculosis not fully complying with their course of treatment (Ali et al., 2019; Godman et al., 2021c). Both areas need addressing going forward to reduce rising AMR. Similarly, polypharmacy can increase healthcare expenditure alongside increase adverse outcomes in patients; consequently, potential patients need careful management once the size of the problem has been calculated (Cho et al.; Kwak et al., 2022). In their study, Cho et al. demonstrated in South Korea that the rate of polypharmacy remained high in the elderly during the past 10 years with the rate of hyper-polypharmacy (currently prescribed 10 or more medicines) had increased, which needs actively addressing (Cho et al.).

In view of these multiple Research Topic, this Research Topic sought to examine the strengths and weaknesses of pharmaceutical policy across countries, and its impact on medicine use and health outcomes to provide future guidance. Overall, 22 papers were published as part of this Research Topic across a wide-variety of areas and countries.

Increasing access to medicines, and enhancing their rational use, are extremely important to countries, especially low- and middle-income countries (LMICs) where there are concerns with the current management of patients (Baumgart et al., 2019; Al-Ziftawi et al., 2021). In their study, Lu et al. discuss the impact of the recent Chinese centralised drug procurement policy, called the ‘4 + 7’ policy, on drug utilisation patterns among public medical institutions (Lu et al.). Lu et al. found that the policy had an appreciable impact with the utilisation of bid-winning medicines increasing from 17.03% to 73.61% of procured medicines. Alongside this, the utilisation of multiple-sourced medicines increased by 67.53% and originators decreased by 26.88% as a result of the policy (Lu et al.). The use-proportion of quality-guaranteed medicines also increased from 56.69% to 93.61% of procured medicines (Lu et al.). Long et al. showed similar findings; however, there were no significant price changes in medicines that were not part of this policy (Long et al.). This mirrors the findings from studies conducted across Europe where the introduction of multiple demand- and supply-side measures can enhance the prescribing of target medicines whilst appreciably reducing expenditure without compromising care (Godman et al., 2021a). The same is not true in countries with limited supply- and demand-side measures (Godman et al., 2021a). In the case of selective serotonin re-uptake inhibitors (SSRIs), Wen et al. showed that the ‘4 + 7’ policy resulted in an appreciable increase in their utilisation (76.7%) alongside decreasing expenditure (3.39%) benefitting patients (Wen et al.). The same has been seen for instance in Scotland where multiple measures resulted in a 73.7% reduction in overall SSRI expenditure between 2001 and 2017 despite their utilization increasing 2.34-fold during this period (Godman et al., 2019).

There have also been similar benefits among hospitals in China as part of a national stewardship policy. Under this policy, key medicines of concern, i.e., those which currently have high prices and utilisation but unconfirmed or limited therapeutic effects, have been subject to close scrutiny with the help of clinical pharmacists (Li et al.). Prior to implementation, there was typically increasing usage and spend on these medicines. However, after implementation in one tertiary hospital with 1,300 beds, Li et al. demonstrated 430 fewer DDDs (defined daily doses) per month in 20 medicines under scrutiny alongside a reduction in overall expenditure of US$4,682 per month in the 19 months post implementation (*p* = 0.003). However in their study, Galimberti et al. failed to show that multiple demand-side measures including feedback reports and online courses failed to appreciably improve GP prescribing in Italy (Galimberti et al.). This contrasts with Sweden where the prescribing of an agreed list of medicines appreciably improved through education and monitoring of prescribing, with a similar situation seen in Sweden and the UK when multiple sourced proton pump inhibitors (PPIs) and statins first became available (Godman et al., 2021a). The differences in findings may be a result of differences in intensity, dissemination and follow-up of the various demand-side measures (Godman et al., 2021a).

There are also concerns with access to medicines and their affordability within the Brazilian healthcare system as it strives to provide universal access (Barbosa et al., 2021; Rocha et al., 2021). In their study, Luz et al. assessed the impact of the ERAF (Estratégia de Regionalização da Assistência Farmacêutica) policy to promote technical cooperation between the State and municipal governments with the aim of improving medicine procurement and distribution. The rationale is to promote the purchasing of high-quality products with reliable suppliers at the lowest-possible prices and transaction costs (Luz et al.). We have seen such procurement activities obtain very low prices for medicines among European countries (Woerkom et al., 2012). However, Luz et al. had concerns that the ERAF policy did not fulfil its goals, with the need to introduce a more sustainable long-term policy to achieve lower prices for medicines as demand grows (Luz et al.). There are similar concerns with the availability of essential medicines within the State of Minas Gerais in Brazil with a need to strengthen funding and public purchasing processes to continue to provide universal healthcare alongside growing demands on available resources (Luz et al.).

On the other hand, the instigation of the Korean Pharmaceutical Information Service (KPIS) to increase transparency in the pharmaceutical supply chain, including the prevention of recalled medicines as well as a decrease in inventory and disposal of out-of-date medicines, resulted in appreciable savings (Kim et al.). Kim et al. calculated the net benefit of the introduction of KPIS at US$571.6 million over 12 years, justifying its introduction (Kim et al.). Such systems can also help monitor possible medicine shortages, which are an increasing concern globally (Acosta et al., 2019). Shortages are more likely to occur with multiple-sourced medicines and older parental medicines where Research Topic of profitability can be a concern, especially where the supply of raw materials is concentrated in only a limited number of manufacturers. In addition, where information systems are lacking to track utilisation and where there are delays in payment (Acosta et al., 2019; Modisakeng et al., 2020; Sarnola et al.). These concerns are leading to pro-active measures across countries to address the situation including planning for shortages with antibiotics (Chigome et al., 2019; Miljković et al., 2020).

Not surprisingly given that new cancer medicines dominate the medicines’ pipeline with over 500 companies actively pursuing the development of new oncology medicines (Godman et al., 2021b), there were an appreciable number of papers examining the cost-effectiveness of different oncology medicines. Such analyses are particularly important in LMICs where there are already Research Topic of cost-effectiveness with existing oncology medicines to treat patients with breast cancer (Al-Ziftawi et al., 2021). In their paper, Zhu et al. documented that toripalimab combined with gemcitabine and cisplatin had a greater chance of being cost-effective compared to camrelizamub combined with gemcitabine and cisplatin or gemcitabine and cisplatin combined from a Chinese payer’s perspective for first-line treatment of patients with recurrent or metastatic nasopharyngeal carcinoma (Zhu et al.). Similarly in their study, Chen et al. demonstrated that capecitabine and oxaliplatin appeared more cost-effective than gemcitabine and oxaliplatin again from the perspective of the Chinese healthcare system (Chen et al.). In their network meta-analysis, Li et al. also found that sintilimab plus biosimilar bevacizumab was cost-effective compared to sorafenib in patients with unresectable hepatocellular carcinoma again from the perspective of the Chinese healthcare system (Li et al.). However, whilst adding atezolizumab to platinum-based chemotherapy as first-line treatment of patients with metastatic urothelial cancer improved survival times, Liu et al. did not find this combination was cost-effective in the Chinese setting (Liu et al.). There were also mixed findings in the systematic review of Chan et al. regarding Poly ADP-Ribose Polymerase (PARP) inhibitors across a variety of cancers (Chan et al.). The authors concluded that in advanced ovarian cancer, PARP inhibitors should be prioritised for upfront maintenance for patients with BRCA mutation or BRCAness at recurrence in view their cost-effectiveness in this situation (Chan et al.). The same was not seen in recurrent maintenance in patients with advanced ovarian cancer even with genetic stratification (Chan et al.).

Sensitivity analyses are particularly important when reviewing potential reimbursement and funding for new cancer medicines given that an increasing number are being launched early with only limited effectiveness and safety data (Godman et al., 2021b). This aspect is discussed further by Bae et al., who urge caution when using immature data especially if this diverts funding away from proven cost-effective technologies and subsequently wastes valuable resources (Pontes et al., 2020; Bae et al.).

In the case of other NCDs, Oh et al. in their study assessed the appropriateness of using EQ-5D-3L and EQ-5D-5L when assessing the role and value (Oh et al.). Unsurprisingly, the pooled utility of patients with asthma declined with worsening control and severity of asthma. The authors concluded that both these health-related quality-of-life instruments are useful when assessing the value of asthma treatments (Oh et al.). Kim et al. showed that easing the criteria for reimbursing statins among patients with type 2 diabetes reduces CV events and their related costs (Kim et al.). This mirrors other studies, demonstrating the need for multiple demand- and supply-side measures to ensure low prices for statins alongside increased use to benefit both patients and the healthcare system (Collins et al., 2003; Godman et al., 2021a). There were though concerns with the improper use of PPIs for patients undergoing stress ulcer prophylaxis in non-critical patients in the study of (Li et al.). The authors concluded that effective intervention strategies including education executed by clinical pharmacists could help address the situation (Li et al.). Effective interventions are also needed to reduce prescription opioid misuse in South Korea highlighted by Kim et al. in their study (Kim et al.).

AMR is an increasing concern across countries exacerbated by appreciable purchasing of antibiotics without a prescription in LMICs (Godman et al., 2021c). Dispensing of antibiotics without a prescription is exacerbated by patient pressure even for self-limiting conditions such as acute respiratory illnesses (Godman et al., 2021c). Consequently, it was encouraging to see in the study of Arshad et al. that the public in Pakistan appeared to be aware of the problems associated with multidrug-resistant pathogens and their responsibilities in helping to reduce AMR (Arshad et al.). In their study, Pradipta et al. found that strengthening ambulatory care facilities, including adequate resources and personnel, is important to ensure patients with TB are adequately managed and adherent to prescribed therapies (Pradipta et al.). Otherwise, resistance will develop, which needs to be avoided to help eradicate TB.

In conclusion, there were a considerable number of papers covering this Research Topic. This demonstrates considerable interest across countries to develop and implement pharmaceutical policies to enhance the rational use of medicines. This includes improve access as well as procurement of medicines. In addition, ensuring value for money within finite resources. A number of initiatives can act as exemplars for the future, building on existing initiatives.

## References

[B1] Acosta A. Vanegas E. P. Rovira J. Godman B. Bochenek T. (2019). Medicine shortages: Gaps between countries and global perspectives. Front. Pharmacol. 10, 763. 10.3389/fphar.2019.00763 31379565 PMC6658884

[B2] Al-Ziftawi N. H. Shafie A. A. Mohamed Ibrahim M. I. (2021). Cost-effectiveness analyses of breast cancer medications use in developing countries: A systematic review. Expert Rev. Pharmacoecon Outcomes Res. 21 (4), 655–666. 10.1080/14737167.2020.1794826 32657174

[B3] Ali M. H. Alrasheedy A. A. Kibuule D. Godman B. Hassali M. A. Ali H. M. H. (2019). Assessment of multidrug-resistant tuberculosis (MDR-TB) treatment outcomes in Sudan; findings and implications. Expert Rev. Anti Infect. Ther. 17 (11), 927–937. 10.1080/14787210.2019.1689818 31689134

[B4] Barbosa M. M. Moreira T. A. Nascimento R. C. Nascimento M. M. Acurcio F. A. Godman B. (2021). Access to medicines in the Brazilian unified health system's primary health care: Assessment of a public policy. J. Comp. Eff. Res. 10 (10), 869–879. 10.2217/cer-2021-0026 34032143

[B5] Baumgart D. C. Misery L. Naeyaert S. Taylor P. C. (2019). Biological therapies in immune-mediated inflammatory diseases: Can biosimilars reduce access inequities? Front. Pharmacol. 10, 279. 10.3389/fphar.2019.00279 30983996 PMC6447826

[B6] Chigome A. K. Matlala M. Godman B. Meyer J. C. (2019). Availability and use of therapeutic interchange policies in managing antimicrobial shortages among South African public sector hospitals; findings and implications. Antibiotics 9 (1), 4. 10.3390/antibiotics9010004 31861923 PMC7168338

[B7] Collins R. Armitage J. Parish S. Sleigh P. Peto R. Heart Protection Study Collaborative Group (2003). MRC/BHF heart protection study of cholesterol-lowering with simvastatin in 5963 people with diabetes: A randomised placebo-controlled trial. Lancet 361 (9374), 2005–2016. 10.1016/s0140-6736(03)13636-7 12814710

[B8] Godman B. Bucsics A. Vella Bonanno P. Oortwijn W. Rothe C. C. Ferrario A. (2018). Barriers for access to new medicines: Searching for the balance between rising costs and limited budgets. Front. Public Health 6, 328. 10.3389/fpubh.2018.00328 30568938 PMC6290038

[B9] Godman B. Egwuenu A. Haque M. Malande O. O. Schellack N. Kumar S. (2021). Strategies to improve antimicrobial utilization with a special focus on developing countries. Life 11 (6), 528. 10.3390/life11060528 34200116 PMC8229985

[B10] Godman B. Fadare J. Kwon H. Y. Dias C. Z. Kurdi A. Dias Godói I. P. (2021). Evidence-based public policy making for medicines across countries: Findings and implications for the future. J. Comp. Eff. Res. 10 (12), 1019–1052. 10.2217/cer-2020-0273 34241546

[B11] Godman B. Hill A. Simoens S. Selke G. Selke Krulichová I. Zampirolli Dias C. (2021). Potential approaches for the pricing of cancer medicines across Europe to enhance the sustainability of healthcare systems and the implications. Expert Rev. Pharmacoecon Outcomes Res. 21 (4), 527–540. 10.1080/14737167.2021.1884546 33535841

[B12] Godman B. Kurdi A. McCabe H. Johnson C. F. Barbui C. MacBride-Stewart S. (2019). Ongoing initiatives within the Scottish National Health Service to affect the prescribing of selective serotonin reuptake inhibitors and their influence. J. Comp. Eff. Res. 8 (7), 535–547. 10.2217/cer-2018-0132 31023070

[B13] Haycox A. (2016). Why cancer? Pharmacoeconomics 34 (7), 625–627. 10.1007/s40273-016-0413-0 27194312 PMC4901109

[B14] IQVIA (2019). The global use of medicine in 2019 and outlook to 2023 - forecasts and areas to watch. Available at: https://www.iqvia.com/insights/the-iqvia-institute/reports/theglobal-use-of-medicine-in-2019-and-outlook-to-2023 .

[B15] Kim Y. Kwon H. Y. Godman B. Moorkens E. Simoens S. Bae S. (2020). Uptake of biosimilar infliximab in the UK, France, Japan, and Korea: Budget savings or market expansion across countries? Front. Pharmacol. 11, 970. 10.3389/fphar.2020.00970 32733238 PMC7363801

[B16] Kwak M. J. Chang M. Chiadika S. Aguilar D. Avritscher E. Deshmukh A. (2022). Healthcare expenditure associated with polypharmacy in older adults with cardiovascular diseases. Am. J. Cardiol. 169, 156–158. 10.1016/j.amjcard.2022.01.012 35168755 PMC9313779

[B17] Kwon H. Y. Godman B. (2017). Drug pricing in South Korea. Appl. Health Econ. Health Policy 15 (4), 447–453. 10.1007/s40258-017-0307-0 28138932

[B18] Luzzatto L. Hyry H. I. Schieppati A. Costa E. Simoens S. Schaefer F. (2018). Outrageous prices of orphan drugs: A call for collaboration. Lancet 392 (10149), 791–794. 10.1016/S0140-6736(18)31069-9 30037734

[B19] Miljković N. Godman B. van Overbeeke E. Kovačević M. Tsiakitzis K. Apatsidou A. (2020). Risks in antibiotic substitution following medicine shortage: A health-care failure mode and effect analysis of six European hospitals. Front. Med. 7, 157. 10.3389/fmed.2020.00157 PMC7235345 32478082

[B20] Modisakeng C. Matlala M. Godman B. Meyer J. C. (2020). Medicine shortages and challenges with the procurement process among public sector hospitals in South Africa; findings and implications. BMC Health Serv. Res. 20 (1), 234. 10.1186/s12913-020-05080-1 32192481 PMC7082963

[B21] Pontes C. Zara C. Torrent-Farnell J. Obach M. Nadal C. Vella-Bonanno P. (2020). Time to review authorisation and funding for new cancer medicines in Europe? Inferences from the case of olaratumab. Appl. Health Econ. Health Policy 18 (1), 5–16. 10.1007/s40258-019-00527-x 31696433

[B22] Rocha W. H. Teodoro J. Assis Acurcio F. Guerra A. A. Jr. Gomes Moura I. C. Godman B. (2021). Influence of pharmaceutical services organization on the availability of essential medicines in a public health system. J. Comp. Eff. Res. 10 (6), 519–532. 10.2217/cer-2020-0259 33739138

[B23] Woerkom M. Piepenbrink H. Godman B. Metz J. Campbell S. Bennie M. (2012). Ongoing measures to enhance the efficiency of prescribing of proton pump inhibitors and statins in The Netherlands: Influence and future implications. J. Comp. Eff. Res. 1 (6), 527–538. 10.2217/cer.12.52 24236472

